# Moral reasoning and moral competence as predictors of cooperative behavior in a social dilemma

**DOI:** 10.1038/s41598-023-30314-7

**Published:** 2023-03-06

**Authors:** Rubén Andrés Miranda-Rodríguez, Iwin Leenen, Hyemin Han, Germán Palafox-Palafox, Georgina García-Rodríguez

**Affiliations:** 1 Universidad Nacional Autónoma de México, Mexico City, Mexico; 2 Educational Psychology Program, University of Alabama, Tuscaloosa, USA Alabama 35487

**Keywords:** Human behaviour, Social behaviour

## Abstract

The level of moral development may be crucial to understand behavior when people have to choose between prioritizing individual gains or pursuing general social benefits. This study evaluated whether two different psychological constructs, moral reasoning and moral competence, are associated with cooperative behavior in the context of the prisoner's dilemma game, a two-person social dilemma where individuals choose between cooperation or defection. One hundred and eighty-nine Mexican university students completed the Defining Issues Test (DIT-2; measuring moral reasoning) and the Moral Competence Test (MCT) and played an online version of the prisoner’s dilemma game, once against each participant in a group of 6–10 players. Our results indicate that cooperative behavior is strongly affected by the outcomes in previous rounds: Except when both participants cooperated, the probability of cooperation with other participants in subsequent rounds decreased. Both the DIT-2 and MCT independently moderated this effect of previous experiences, particularly in the case of sucker-outcomes. Individuals with high scores on both tests were not affected when in previous rounds the other player defected while they cooperated. Our findings suggest that more sophisticated moral reasoning and moral competence promote the maintenance of cooperative behaviors despite facing adverse situations.

## Introduction

The relationship between morality and cooperation provides a fundamental perspective on the unselfish aspects of human nature^1–3^. From an evolutionary point of view, the ability to organize large societies through cooperation, division of labor, and reciprocal altruism (i.e., ultra-sociality)^4,5^ requires a tendency to sacrifice individual interests for the sake of others’ welfare^6^. These behaviors encompass a sense of cooperation and morality that has been fundamental for human social development^7^. Today, the Covid-19 pandemic has affected all human beings and made it essential to organize for mutual protection in the face of the harmful consequences for humanity. This organization requires cooperation^8^.

This relationship between morality and cooperation has been studied extensively and under a variety of paradigms. Early attempts to study the relation between attitudes towards certain moral principles or standards (like respect, justice, benevolence; as measured through self-report questionnaires) and specific cooperative behaviors found, as an overall result, that these behaviors are difficult to predict from such global attitudes^9^ (which confirms the general finding in social psychology known as the attitude-behavior inconsistency^10^). On the other hand, studies that focused on specific moral constructs have shown that inducing moral emotions like gratitude and guilt increases cooperation^11–14^ and that moral disengagement correlates negatively with cooperative behavior^15,16^.

Thus, general attitudes towards moral standards or principles are not useful to predict cooperative behavior while some specific emotions can modulate it, suggesting that simple valuation judgments do not capture adequately the response of the affect system and that held/crystallized beliefs by themselves are a weak trigger for behavior. In this study we propose that a more active process of valuation that relies on the capacity to reason morally could be a more useful predictor of cooperative behavior. Lawrence Kohlberg, arguably the most well-known author in the field of morality, included in his theory the construct of *moral capacity*, which he defined as the ability to judge and act based on moral judgments; thus, moral capacity is observed at the level of reasoning. In particular, Kohlberg’s theory assumes that the development of moral capacity passes through six stages or moral orientations^17,18^: (1) heteronomous morality, in which reasoning is based on physical consequences and the judgment of others, normally some authority; (2) instrumental individualistic morality, where reasoning is based on solely personal needs and desires; (3) normative interpersonal morality, in which reasoning is done according to the expectations of the social environment; (4) the morality of the social system, in which reasoning is based on compliance with established norms, rules, or laws; (5) the morality of the social contract, where reasoning is based on human rights and the search for agreements that benefit everyone; and (6) the morality of universal ethical principles, in which one reasons according to one's own conscience about certain universal principles, such as well-being and justice. Stages 1 and 2 are classified as pre-conventional, stages 3 and 4 as conventional, and stages 5 and 6 are known as post-conventional stages. Studies based on this taxonomy report significant relations between these levels of reasoning and behavioral outcomes, including cooperation. For example, McNamee^19^ showed that higher levels of moral reasoning increase the likelihood of prosocial behavior.

Rest and colleagues suggested a revision of Kohlberg’s original model and proposed an update, which is nowadays known as the neo-Kohlbergian model^20–23^. They argued that it is necessary to move from a hard model that assumes moral reasoning as an immutable ladder of stages to a soft model of cognitive schemes. In this sense, they proposed three cognitive schemes that represent moral reasoning: (1) personal interests, in which moral judgment depends on individual benefits; (2) maintaining norms, in which moral judgment depends on social rules; and (3) postconventional reasoning, in which moral judgment depends on the search for global well-being. This conceptual change allows us to understand the development of *moral reasoning* in terms of the probability of using one or another scheme for problem solving in specific contexts.

The neo-Kohlbergian perspective has been implemented in the Defining Issues Test (DIT) and, as such, has been widely used in the field to assess the development of moral reasoning^24,25^. The latest version of this test (DIT-2) presents five moral dilemmas to the respondent (see, Materials and Instruments in the Method section for further details) and defines the N2-index which quantifies, on a scale from 0 to 100, the ability of a person to justify their decisions based on a reasoning that adheres more to the post-conventional scheme, rather than to the personal interest scheme^21^.

In an alternative approach to moral reasoning proposed by Lind^26,27^, the construct of *moral competence*, defined as the ability to solve problems based on moral principles through dialogue and deliberation instead of violence and deceit, is central. To measure moral competence and use the construct in applied research, Lind developed the Moral Competence Test (MCT), which like the DIT presents several moral dilemma scenarios to the respondents and requires them to rate the validity of each of a number of arguments for each dilemma^28,29^. The main score derived from the MCT is the C-score (Competence), which quantifies on a scale of 0–100 the respondent’s consistency when evaluating judgments corresponding to each of the six stages in Kohlberg’s model, both across different dilemmas and across arguments either in favor or against a particular decision in the context of a dilemma ^27^.

In a study that assessed the relationship between moral competence and cooperation in a competitive condition where participants that gained the most profits would receive the best prizes, Perry and Clough^30^ found that moral competence was positive correlated with cooperative behavior; however, it was not a statistically significant predictor and the question remains whether there is a relationship between moral competence and cooperative behavior in a non-competitive experimental situation.

Although related, moral reasoning and moral competence are different constructs. While the former focuses on the cognitive process and reasoning that underly moral judgments and their development, the latter is more concerned about the decision-making aspects behind moral behavior. Moral competence is more about whether one is able to take different perspectives and to identify shared moral principles, independent from one’s own moral reasoning developmental level (viz., the developmental stage or cognitive scheme adopted). In particular, whether one is capable of applying their moral schema consistently across different dilemmas and situations becomes the main focus of moral competence. This difference between the moral reasoning and moral competence model also explains why their associated questionnaires (DIT-2 and MCT, respectively) use different scoring systems. The C-score derived from the MCT, in contrast to the N2-index of the DIT-2, takes only the degree of consistency into account and ignores whether the moral judgments are based on pre-conventional, conventional, or post-conventional stages; on the other hand, Lind shows evidence for a moderate positive correlation between both constructs: Individuals with higher moral competence tend to accept arguments referring to highly sophisticated, post-conventional reasoning and reject pre-conventional arguments ^31^.

To our knowledge, the predictive power of these two measures in relation to cooperative behavior has not yet been studied. Han et al.^28^, who evaluated the effect of moral reasoning and moral competence on the response time in moral dilemmas, suggested that researchers should consider different types of social contexts and dilemmas in future research for understanding how moral reasoning and moral competence predict specific behaviors. In this study, we take up this challenge and examine the relationship between moral reasoning, moral competence, and cooperative behavior in a specific social dilemma, namely the prisoner’s dilemma game (PDG), a widely used experimental paradigm to study conflict resolution in social psychology^32–34^.

**Figure 1:**
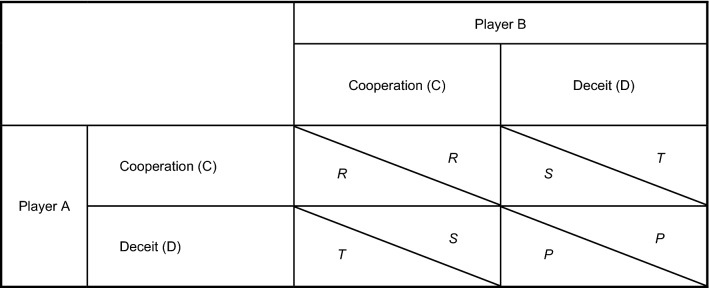
Pay-off matrix in the Prisoner's Dilemma game, showing the consequences (gains or losses) in function of the decisions (cooperation (C) or deceit (D)) by both players. The symbols *S*, *P*, *R*, and *T* denote the benefits in each possible condition for Player A (below the diagonal) and Player B (above the diagonal). For the game to be a social dilemma, it is required that *S* < *P* < *R* < *T* and 2*R* > *S* + *T*.

In the standard version of this two-person game, where the collective interest is typically in conflict with the private interest, both players make a choice between cooperation (C) or defection (D) independently (i.e., without communication). Figure 1 shows the general format of the payoff matrix associated with the game, where the payoffs for Player A (below the diagonal) as well as for Player B (above the diagonal) are a function of the choices (C or D) made by each of the players. The symbols *S*, *P*, *R*, *T* stand for the “sucker” (the payoff received by cooperating while the other defects), “punishment” (the payoffs when both players defect), “reward” (when both players cooperate), and “temptation” (when one defects and the other player cooperates) outcomes, respectively. In most studies^35–37^, as in the present research, the payoffs are operationalized in terms of monetary rewards or costs (unlike in the original Prisoner’s Dilemma, which presents the rewards and costs in terms of years in prison for two prisoners suspected of a crime). In general, payoffs may be negative (representing losses) or positive (representing gains) and may differ between both players (i.e., in asymmetric PDGs). The current study employs a symmetric PDG, where the S, P, R, and T payoffs are positive and identical for both players. The game is a social dilemma if the following two conditions are satisfied^38^:

1. *S* < *P* < *R* < *T*. This restriction implies that, on the one hand, it is *individually* rational to choose defection over cooperation since for each player benefits are larger for D than for C, irrespective of the choice of the other player (i.e., *T* > *R* as well as *P* > *S*); on the other hand, *collectively,* joint cooperation is more beneficial than joint defection (i.e., *R* > *P*).
2. 2*R* > *T* + *S*. As an additional restriction, this guarantees that the *joint* benefit is higher when both players cooperate as compared to any other combination.

In this study, we explored how moral reasoning^17,18^ and moral competence^26,27^ jointly predict behavioral outcomes, particularly moral decision making operationalized as cooperative behavior in the PDG. We used an *iterated stranger design* for the PDG (i.e., individuals play the PDG for several rounds, once against each other individual of a group of 6–10 persons). We examined how variations in cooperative behavior in this social dilemma were predicted by the N2-index of the DIT-2^21^, and the C-score of the MCT^27^.

Given the definition and the nature of moral reasoning and moral competence, we hypothesized that (a) more sophisticated moral reasoning (in terms of a higher N2-index on the DIT-2) would be associated with a *higher likelihood of cooperation* represented by the individual’s overall cooperation level in the PDG; and (b) stronger moral competence (in terms of a higher C-score on the MCT) would be associated with a higher *consistency* in the choice of either cooperation or defection across different occasions/rounds, that is: individuals with a stronger moral competence would be more likely to behave similarly across different situations based upon their moral principles.

## Results

### Descriptive statistics

**Figure 2:**
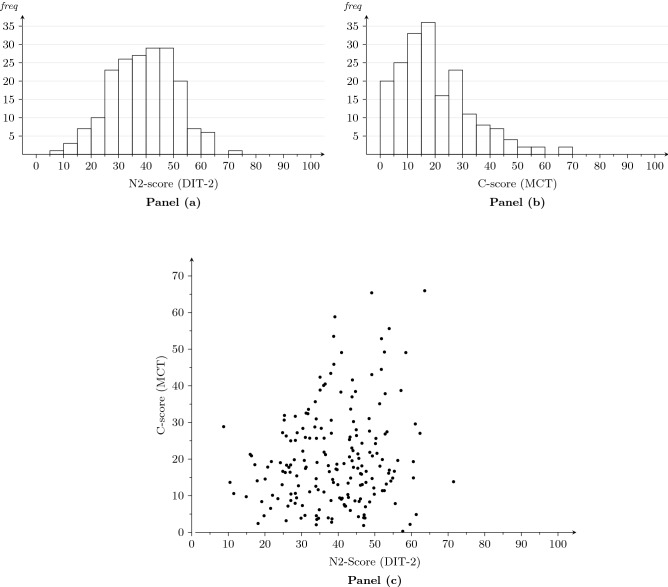
Panel (**a**): distribution of the scores on the N2-index; Panel (**b**): distribution of the C-scores; Panel (**c**): scatter diagram of the scores on the N2-index and C-scores.

Panels (a) and (b) of Fig. 2 show the frequency distributions of the N2-index and the C-score. The means are: 39.2 (*SD* = 11.8) for the N2-index and 20.1 (*SD* = 13.1) for the C-score, while the medians are 39.6 and 17.8, respectively. Panel (c) represents the bivariate distribution of both scores through a scatter diagram; the Pearson correlation equals 0.17 (Fisher’s *z*(187) = 0.17, *p* = 0.02, 95% confidence interval [CI] = [0.03,0.31]).

**Figure 3:**
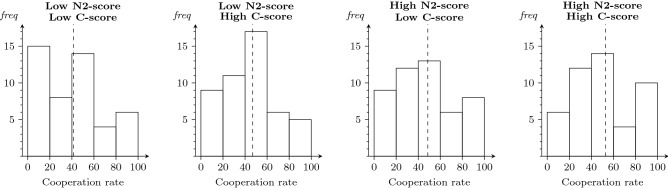
Distribution of individuals’ cooperation rate (percentage of cooperation across all rounds) in function of their N2-index and C-score below or above the median. The dashed line indicates the mean cooperation rate.

With respect to the behavior in the PDG, we calculated, for each participant, their cooperation rate (i.e., the percentage of cooperation) across all iterations of the game. Figure 3 shows the distribution of these rates in four groups of participants, classified by their score on the N2-index and C-score, i.e., below or above the sample median. Although the mean cooperation rate turns out to be higher among participants with high scores on the N2-index and high C-scores, this tendency is not statistically significant (see below).

### Inferential statistics

We present the results of the inferential analyses based on the statistical model described in the Method section in three stages. First, we examined how experiences in the previous rounds affected the probability of cooperation in the current round of the PDG. Including these prior experiences as a first step in the model is motivated by the results from other studies that showed that they strongly determine the behavior in the PDG^39^ so that other effects may be masked or attenuated if these prior experiences are omitted from the model. The column under Model 1 in Table 1 shows the estimated effect of having experienced one sucker (S-History), punishment (P-History), reward (R-History), and temptation (T-History) outcome in one of the previous rounds. Note that, while in Table 1 results are presented on the log-odds scale that underlies the logistic regression model, we’ll report here the results equivalently as odds ratios (OR). The results show that the probability of cooperation decreases for each sucker (OR 0.84, CI [0.74,0.96]), punishment (OR 0.70, CI [0.60,0.81]), and temptation (OR 0.66, CI [0.56,0.76]) outcome in the previous rounds, whereas a prior reward outcome makes cooperation more likely (OR 1.19, CI [1.05,1.34]). Importantly, the effects are modeled as additive on the log-odds scale, which implies that a (sucker, punishment, reward, or temptation) experience has the same effect on cooperation regardless of whether the experience occurred in the preceding round or in an earlier round (e.g., at the start) of the experiment. For example, if a participant had a sucker and temptation experience during the first two rounds, the probability of cooperation in the third round is 0.17 + 0.42 = 0.59 units lower on the log-odds scale (i.e., OR 0.55, CI [0.45,0.67]) as compared to the first round in the experiment, regardless of whether the sucker or the temptation experience was the most recent.

**Table 1:** Parameter estimates of the mixed-effects logistic regression models to predict cooperative behavior in the prisoner’s dilemma.

	Model 1	Model 2	Model 3
*Fixed effects*			
Intercept (mean)	0.40** (0.113)	0.41** (0.115)	0.44** (0.122)
History variables			
S-History	− 0.17* (0.067)	− 0.19** (0.068)	− 0.68** (0.152)
P-History	− 0.35** (0.076)	− 0.34** (0.076)	− 0.40** (0.149)
R-History	0.17** (0.062)	0.16* (0.063)	0.26* (0.109)
T-History	− 0.42** (0.078)	− 0.41** (0.079)	− 0.45** (0.143)
Moral questionnaires			
Z(N2-Score) (DIT)		0.11 (0.083)	0.04 (0.111)
Z(C-Score) (MCT)		0.04 (0.083)	− 0.04 (0.107)
Interaction effects			
S-History × High-DIT-2			0.30* (0.149)
P-History × High-DIT-2			− 0.00 (0.158)
R-History × High-DIT-2			− 0.08 (0.129)
T-History × High-DIT-2			0.16 (0.160)
S-History × High-MCT			0.38* (0.150)
P-History × High-MCT			0.18 (0.160)
R-History × High-MCT			− 0.14 (0.130)
T-History × High-MCT			0.02 (0.164)
*Variance parameters*			
Intercept (variance)	0.50** (0.241)	0.56** (0.256)	0.79** (0.312)

Estimates are reported on the log-odds scale. Model 1 includes only the history variables as predictors; Model 2 includes both the history variables and the scores on the moral questionnaires; Model 3 adds the interactions. Z(N2-Score) and Z(C-Score) are the standardized N2-Score and C-Score, respectively, based on the sample means and variances. High-DIT-2 and High-MCT are binary variables with a value of 1 for N2-Scores and C-Scores above their respective median. The standard error associated with the estimated effect is shown between parentheses. *(*p* < 0.05) and **(*p* < 0.01) indicate that the estimate is significantly different from 0.

We checked the latter assumption of additivity in a subsequent analysis, which separated the effect of the most recent experience (i.e., in the preceding round) from the effect of experiences in earlier rounds. The results show a tendency that the most recent experience has a larger effect as compared to the experience in rounds prior to the preceding round. However, as this tendency turned out to be nonsignificant (*F*(4,1172) = 2.18, *p* = 0.07), we maintained the initial model with the assumption of additivity.

At the second stage (Model 2), we added the N2-index (from the DIT-2) and the C-score (from the MCT) as main effects to the predictors in Model 1. The results (see the column under Model 2 in Table 1) show that both these variables have a nonsignificant effect on the probability of cooperation. Translated to odds ratios, these results imply that an increase of one standard deviation in the N2-index multiplies the odds of cooperation by 1.11 (CI [0.95,1.31]); likewise, the odds ratio equals 1.04 (CI [0.89,1.23]) for a one standard deviation increase of the C-score. In sum, these results suggest that the responses on the DIT-2 and the MCT do not directly predict cooperation in the PDG.

**Figure 4:**
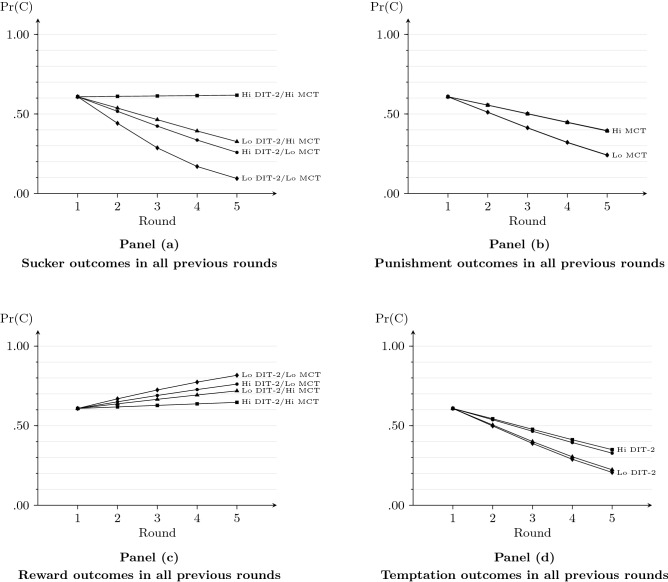
Predicted probability of cooperation as a function of outcomes in previous rounds of the prisoner’s dilemma game and high versus low scores on the DIT-2 and MCT.

Finally, we added the interactions between the history variables on the one hand and the N2-index and C-score on the other hand (Model 3). To this end, we dichotomized the latter variables creating a group variable for both moral reasoning and moral competence that categorizes their scores into low versus high level (i.e., scores above or below the sample median). The N2-index as well as the C-score group categories significantly interacted with sucker experiences in the previous rounds. In particular, if a participant has both low moral reasoning and low moral competence (i.e., both the N2-index and the C-score below their respective medians), then a sucker outcome halves the odds of cooperation (OR 0.51, CI [0.38,0.69]); with the N2-index above and the C-score below the median, this odds ratio increases to 0.69 (CI [0.53,0.89]) and with the C-score above and the N2-index below the median, the odds ration becomes 0.75 (CI [0.59,0.95]). Moreover, as these moderator effects are modeled to be additive, these results imply that above-average scores for *both* the N2-index and the C-score neutralize the negative effect of sucker experiences in previous rounds (OR 1.01, CI [0.81,1.26]). That is, in individuals with *both* sophisticated moral reasoning and strong moral competence, the probability of cooperation in the PDG does not change when they are confronted with the adverse situation of a sucker outcome in the previous rounds of the game (with other players). Panel (a) of Fig. 4 shows, by way of example, the predicted probability of cooperation in each of rounds 1 to 5 in the hypothetical case that a participant experienced “Sucker” outcomes in *all* previous rounds, classified by their N2-index and C-score (being below or above their respective medians). As explained above, if both scores are above the median, the probability of cooperation remains constant and unaffected by the experience of sucker outcomes in previous rounds. Interestingly, the N2-index and C-score do not significantly moderate the effect of experiences other than the sucker experience as shown in the last column of Table 1 and in panels (b) to (d) of Fig. 4.

## Discussion

Cooperative behavior is an unselfish manifestation of human social nature^1–3^. It implies refraining from a higher individual benefit to open the possibility of an increased collective benefit, which only becomes real, though, if others respond in a reciprocal way^40^. However, individuals —even those who are generally oriented to act in favor of social benefit— are likely to modify their behavior and turn to a strategy that maximizes individual gains and/or minimize individual losses, once they realize that their cooperative efforts are not being reciprocated^41,42^. Previous studies have confirmed that reciprocity is required for sustained cooperative behavior among both humans and animals^43–46^. The results of our study are consistent with these findings as we demonstrated that first, experiencing non-reciprocity to cooperative behavior (viz., having a sucker experience in the PDG) strongly decreased the likelihood of repeated cooperation; and second, reciprocity to cooperation (i.e., a reward outcome) enhanced the probability in the subsequent rounds.

In this respect, it is important to stress that we used an *iterated stranger* design, which implies that each player only interacts once with each other player in the group without knowing whether they cooperated in the previous rounds playing with others. Contrary to the case of repeatedly interacting with the *same* player, no strategy based on direct reciprocity (e.g., tit-for-tat strategy)^44^ can be reasonably applied. Also indirect-reciprocity strategies, where information about previous interactions with other persons can be taken into account during the current interaction, or the behavior in the current interaction may be considered in future interactions (e.g., based on reputation)^47^, are ruled out. Given that such information is not available, one may expect, from the perspective of evolutionary game theory, that in this study the dominant choice in the PDG is defection^48^. However, other studies have shown that even in this situation cooperation is not uncommon, with cooperation rates close to 40%^49,50^.

Furthermore, in an iterated stranger design there is no reason to change strategies (from cooperating to defecting or vice versa) during the game. However, our study shows that the effect of reciprocity and non-reciprocity, as a response to cooperation experienced in previous interactions, transfers to other participants with whom players have no shared history or about whom no information is available nor will be shared with others in the future. Although in conflict with the expectations based on evolutionary game theory, this result may be accounted for by the principle of reinforcement in behaviorist psychology, which predicts that a response will be repeated if it turned out to be successful at a previous occasion, whereas an unsuccessful one will not be^51,52^.

Indeed, in the context of the PDG, a player who opts for the noncooperative choice is reinforced independently of the choice by the other player (i.e., for both the punishment outcome and the temptation outcome) given that they compare their actual gains with the gains they would have won if they had chosen to cooperate; that is, in either case, their choice to defect is reinforced. Furthermore, in the case of a sucker experience, the player most likely reconsiders their choice to cooperate as unsuccessful and, hence, cooperation is not reinforced. Only when both players cooperate (reward outcome), the cooperative behavior is considered a success and will be reinforced for the subsequent rounds. In sum, our study confirms previous results, by showing that (1) reinforcement of cooperative behavior requires an initial collaborative attitude as well as a positive response by others and (2) cooperation or defection is likely to persist in subsequent interaction, except when cooperation meets with defection by the other player.

We also examined how one’s moral reasoning development and competence, as assessed by the DIT-2 and MCT, respectively, predicts cooperative behavior in the PDG, which was the main focus of our study. Interestingly, the main effect of moral reasoning development or moral competence does not significantly predict cooperation. Perhaps, moral reasoning development or competence per se does not fully explain one’s tendency of cooperation during the PDG. However, we found the significant negative interaction effects between, on the one hand, sucker experiences in previous rounds, and (categorized) moral reasoning development and moral competence, on the other. Among participants with high moral reasoning and moral competence, such negative, unsuccessful outcomes did *not* negatively affect the probability of cooperation. Instead, they sustained their collaborative attitude even after experiencing egoistic responses from other participants in previous rounds. They continued their (cooperative or defective) behavior in the PDG not only in the cases when they had defected or the other player had cooperated, but also when their cooperative behavior had not been reciprocated by their counterpart.

Several previous studies in moral psychology may provide useful insights about why moral reasoning and competence moderated the relationship between experiences in the prior rounds and cooperation in the current round during the PDG. Previous studies employing the DIT showed that participants with more sophisticated, postconventional moral reasoning, were more likely to show moral behavior in a consistent manner^53,54^. Furthermore, students with more advanced moral reasoning are more likely to be tolerant of outgroup members with different political and cultural views, who may be perceived as a threat to them^55^. The significant moderating effect of moral reasoning on sustained cooperation after sucker experiences is consistent with these trends reported in these previous studies: Participants with more sophisticated moral reasoning might be more consistently tolerant of counterparts, even when they have been sucked by them.

Han et al.^28^ showed a moderating effect of high moral competence on behavior, in the sense that higher moral competence positively moderated the association between postconventional moral reasoning and consistent responses to moral dilemmas. They suggested that such a great consistency in moral decision making found among participants with sophisticated moral reasoning and strong moral competence is attributable to the presence of better cognitive capacities.

### Limitations and future directions

Moral principles do not work in abstract but are applied on and interact with relevant aspects of the situation. In the particular case of the PDG, it has been shown, for example, that the probability of cooperation is affected by the structure of the pay-off matrix (e.g., whether or not it is symmetric, whether the outcomes are gains or losses, the height of these gains and losses^39^), and even the labels given to the two possible choices^34^. Future research may aim at generalizing the results of the current study investigating the hypothesis that, in persons with high levels of moral reasoning and/or competence and that their probability of cooperation is less affected (as compared to other persons) by the aspects of the situation, and particularly, that their cooperation is more steady despite adverse experiences.

It is also important to note that in our analyses, experiences with other players in previous rounds of the PDG were modeled as single additive effects. This might suggest that on average (see Model 1 in Table 1), the negative effect of a sucker experience can be revoked or alleviated by the positive effect of a reward and that the reinforcing effect of a punishment on non-cooperation is commensurate with about twice the reinforcing effect of a reward on cooperative behavior. As a possible limitation of our study, recent experiences and experiences that go back further in time are weighted equally in the statistical analysis (e.g., a sucker experience in the previous round has the same effect as one in the first round). A larger sample would be required to model such differential effects.

Moreover, other types of differential effects would be interesting to explore in future research. For example, psychology students are overrepresented in the sample of this study (as in much empirical research in Psychology; see the Participants subsection below) and given that psychology students usually have a somewhat different social profile as compared to students from other careers, results in the area of moral reasoning, moral competence, and prosocial behavior may be biased. Likewise, the effects reported in this study may differ between men and women, between students from public and private universities, among different age groups, and so on. Again, a larger sample size in future research may shed a light on whether the reported results hold in general or whether they are more pronounced in some subpopulations. Further research on the conditions when cooperation occurs, together with a better understanding of the moral constructs that moderate or mediate this behavior, may lead to new insights and more accurate theoretical models of cooperative behavior in social dilemmas.

### Conclusion

Our study adds to the body of evidence that more advanced moral reasoning and moral competence are associated with consistent and tolerant behavioral tendencies in social dilemmas. To the best of our knowledge, our study is the first that investigated the simultaneous effect of moral reasoning and moral competence on cooperative behavior and shows that both, in a similar but independent way, reduce the effect of adverse prior experiences on cooperation.

## Methods

### Participants

One hundred and eighty-nine undergraduate and postgraduate students from different majors at Mexican universities participated in this study. Participants’ age ranged from 17 to 46 years (mean = 21.7; SD = 4.4), 74% were women, and all but six were from public universities. Most participants were majoring in health sciences (62% psychology, 7% medicine, 7% other health sciences), although other majors were represented as well (e.g., 11% international affairs and 6% engineering). The authors and collaborators directly recruited the participants either via verbal advertisement in classes or on the social media pages (i.e., webpage, Facebook, Twitter) of the Psychology Faculty at the National Autonomous University of Mexico. Participation was completely voluntarily and each participant was compensated with a small monetary reward ranging from $100 MXN to $270 MXN (i.e., US $5 to US $12.5), as a result of their participation in the PDG. Informed consent was obtained from each participant during the first phase of their participation.

### Materials and instruments

During the first phase of the research, the participants were asked to complete a Google Form containing the Spanish version of the DIT-2^22^. This test consists of five hypothetical moral dilemmas. For each dilemma, participants were requested to make a behavioral decision and, subsequently, were asked to rate the importance of 12 justifications for this decision presented each one in a separate statement with responses anchored to a five-point Likert scale (1 = Extremely important, 2 = Very important, 3 = Some important, 4 = Little important, 5 = Not important). Moreover, at the end of each dilemma, participants were asked to choose and rank-order the four most important statements. We used the N2-index to assess one’s moral reasoning development. This score is considered the most valid single score that locates the respondent on a continuum of moral reasoning development, ranging from the personal-interest schema to the post-conventional schema.^21^ Completed DIT-2 forms were scored by the Center for the Study of Ethical Development (University of Alabama).

During the second phase, the participants were invited to connect to the LIONESS Lab website (https://lioness-lab.org/). On this webpage, researchers can program and run interactive online experiments with multiple participants^56,57^. We designed three multiplayer scenarios, for a group of six, eight, and ten players, respectively, in which each participant plays one round of the PDG with each of the other participants. In each round, the software formed pairs of participants and asked each participant to select one of two options, A or B, while representing the pay-off matrix (constant across rounds) with the possible gains in function of the selection of both players. After confirming their decision by clicking the “Continue” button, they were informed about the decision made by the other player and the resulting gain in the round.

Each scenario was programmed such that the total compensation ranged from $100 MXN to $270 MXN depending on the decisions made by participants and their counterparts in the repeated rounds. In the case of 10-player sessions (9 rounds), during each round, one could earn *S* = *$12 MXN, P* = *$18 MXN, R* = *$24 MXN,* and *T* = *$30 MXN* (see Fig. 1). For 8-player sessions (7 rounds), payments per round were *S* = *$15 MXN, P* = *$20 MXN, R* = *$30 MXN,* and *T* = *$35 MXN*. Finally, for 6-player sessions (5 rounds), the possible earnings per round were *S* = *$20 MXN, P* = *$30 MXN, R* = *$40 MXN,* and* T* = *$50 MXN*.

After the experiment, participants were asked to respond, in an individual and private manner, a second Google Form with a few additional questions about the experiment (e.g., whether they had previously participated in a similar dynamic and whether they knew other people in the same PDG session). Then, they completed the Spanish version of the MCT^58^. The MCT presents two hypothetical moral dilemmas, where the actors already made their behavioral decision. Per dilemma, a total of 12 statements was presented, six corresponding to the six stages of the original Kohlbergian model that are in favor of the decision made and the other six against the decision. Then, participants were asked whether they accepted or rejected each statement, responding using a Likert scale from − 4 (“I totally reject this statement”) to + 4 (“I totally accept this statement”). The C-score, ranging from 0 to 100, was then calculated, with higher scores representing stronger moral competence from Lind’s perspective. The C-score is conceptually equivalent to the *R*^2^-statistic from a one-way analysis of variance of the respondent’s 24 Likert-responses with the level of moral reasoning represented by the statements in terms of six stages as the independent variable.

### Procedure

The current study received endorsement from the ethical review board of the Faculty of Psychology at the National Autonomous University of Mexico and was carried out in full compliance to the American Psychological Association’s *Ethical Principles of Psychologists and Code of Conduct*, and the *Ethical Code* of the Mexican Society of Psychology: Participation was voluntary, informed consent was obtained from all participants, and no sensitive or personal information beyond data necessary for identification within the study was solicited.

While completing the first Google Form (see above), participants were asked to provide their email address so that we could contact them to schedule the in-person experimental session. According to participants’ availability, a group of 6 to 12 students was invited to each online experimental session. In the case that an odd number of participants showed up in the session, a confederate joined the session, because two participants were supposed to be paired in each round. The confederate responded randomly (with equal probabilities for options A and B) in each round.

During the PDG sessions, participants joined a Zoom meeting, where the experimenter explained further details about the experiment and answered questions about the game. Then, through the Zoom chat, the participants were provided with a link to access a designated PDG scenario available via the LIONESS Lab. Before starting the game session, the participants were requested to provide their email address and determine their nickname. The nickname was used to identify each player during the session.

In each round, two windows were presented. In the first window, with title “Decision *x*” (with *x* being a number between 1 and 9 denoting the round), a table with the possible combinations of choices and associated profits (like Table 1) according to the PDG conditions was presented. At the bottom of the window, participants were asked to make their decision by clicking on one or two options (A or B) and confirm the decision. Then, the second window titled “Result *x*” appeared and demonstrated the decisions by both players and the profit gained in the current round as well as the total profit so far. After clicking the “Continue” button in this window, the participants proceeded to the next round until all players had played once against each other individual in the session.

At the end of the experiment, the participants were provided with a link to the second Google Form, which was supposed to be completed after the end of the Zoom meeting. On the last page of this Google Form, the participants provided bank account information that we needed to wire their compensation.

### Data analysis

As the main analysis in the present study, we performed mixed-effects logistic regression analysis with dependent variable *Y*_*ij*_, which assumes a value of 1 if individual *i* cooperated (i.e., chose Option B) in round *j* of the experiment and 0 otherwise. The predictors entered the model in three separate steps. First, the history of cooperation/deceit by each participant and their counterpart in the rounds prior to round *j* was entered. For this, four variables were employed: *S-History*, *P-History*, *R-History*, and *T-History*, which represent the number of previous rounds in which the individual participated and that resulted in a sucker (*S*), punishment (*P*), reward (*R*), and temptation (*T*) outcome, respectively (see Table 1). Second, we entered the N2-index and C-score as predictors to the model. For a better interpretation, these continuous variables were standardized. Third, we added interaction effects between the history variables and the categorized N2-index and C-score. We created eight new variables (*S-History* × *High-DIT-2*), (*P-History* × *High-DIT-2*), (*R-History* × *High-DIT-2*), (*T-History* × *High-DIT-2*), (*S-History* × *High-MCT*), (*P-History* × *High-MCT*), (*R-History* × *High-MCT*), and (*T-History* × *High-MCT*), which are the product of the respective history scores defined above and the variables *High-DIT-2* and *High-MCT*. The *High-DIT-2* and *High-MCT* are binary variables with values of 1 if the N2-index and the C-score, respectively, are above the respective median score, and 0 otherwise. To account for the nested structure of the data (with observations from multiple rounds nested within a single participant), we specified the intercept as random, varying across participants. These regression analyses were conducted with the PROC GLIMMIX procedure of SAS 9.4^59^.

## Data Availability

The data that were collected and analyzed during the current study are available from the corresponding author on reasonable request.

## References

[1] Curry OS, Jones Chesters M, van Lissa CJ (2019). Mapping morality with a compass: Testing the theory of ‘morality-as-cooperation’ with a new questionnaire. J. Res. Pers..

[2] Korthals M (1992). Morality and cooperation. J. Moral Educ..

[3] Hamlin JK (2013). Moral judgment and action in preverbal infants and toddlers. Curr. Dir. Psychol. Sci..

[4] Christens BD (2020). Ultrasociality and intersubjectivity. Am. J. Commun. Psychol..

[5] Gowdy J, Krall L (2016). The economic origins of ultrasociality. Behav. Brain Sci..

[6] Trivers RL (1971). The evolution of reciprocal altruism. Q. Rev. Biol..

[7] Tomasello M, Vaish A (2013). Origins of human cooperation and morality. Annu. Rev. Psychol..

[8] van Bavel JJ (2020). Using social and behavioural science to support COVID-19 pandemic response. Nat. Hum. Behav..

[9] Clark CB (2017). A behavioral economic assessment of individualizing versus binding moral foundations. Pers. Ind. Dif..

[10] Azjen I, Fishbein M, Lohmann S, Albarracín D, Albarracín D, Johnson BT (2018). The influence of attitudes on behavior. The Handbook of Attitudes.

[11] Dal Bó E, Dal Bó P (2014). “Do the right thing:” The effects of moral suasion on cooperation. J. Public Econ..

[12] DeSteno D, Bartlett MY, Baumann J, Williams LA, Dickens L (2010). Gratitude as moral sentiment: Emotion-guided cooperation in economic exchange. Emotion.

[13] Ketelaar T, Tung Au W (2003). The effects of feelings of guilt on the behaviour of uncooperative individuals in repeated social bargaining games: An affect-as-information interpretation of the role of emotion in social interaction. Cogn. Emot..

[14] de Hooge IE, Zeelenberg M, Breugelmans SM (2007). Moral sentiments and cooperation: Differential influences of shame and guilt. Cogn. Emot..

[15] Gülseven Z, Yu MVB, Zarrett N, Vandell DL, Simpkins SD (2021). Self-control and cooperation in childhood as antecedents of less moral disengagement in adolescence. Dev. Psychopathol..

[16] Ogunfowora B (2021). The impact of team moral disengagement composition on team performance: The roles of team cooperation, team interpersonal deviance, and collective extraversion. J. Bus. Psychol..

[17] Kohlberg L, Hoffman LW, Hoffman ML (1964). Development of moral character and moral ideology. Review of Child Development Research.

[18] Kohlberg L, Kohlberg L (1987). The development of moral judgment and moral action. Child Psychology and Childhood Education: A Cognitive-Developmental View.

[19] McNamee S (1977). Moral behaviour, moral development and motivation. J. Moral Educ..

[20] Rest J, Narvaez D, Bebeau M, Thoma S (1999). A neo-Kohlbergian approach: The DIT and schema theory. Educ. Psychol. Rev..

[21] Rest J, Thoma SJ, Narvaez D, Bebeau MJ (1997). Alchemy and beyond: Indexing the Defining Issues Test. J. Educ. Psychol..

[22] Rest JR, Narvaez D, Thoma SJ, Bebeau MJ (2000). A Neo-Kohlbergian approach to morality research. J. Moral Educ..

[23] Thoma SJ (2014). Measuring moral thinking from a neo-Kohlbergian perspective. Theory Res. Educ..

[24] Bebeau MJ (2002). The Defining Issues Test and the four component model: Contributions to professional education. J. Moral Educ..

[25] Schlaefli A, Rest JR, Thoma SJ (1985). Does moral education improve moral judgment? A meta-analysis of intervention studies using the Defining Issues Test. Rev. Educ. Res..

[26] Lind G (2011). Moral competence and the democratic way of living. Eur. J. Psychol..

[27] Lind G (2016). How to Teach Morality: Promoting Deliberation and Discussion, Reducing Violence and Deceit.

[28] Han H, Dawson KJ, Thoma SJ, Glenn AL (2020). Developmental level of moral judgment influences behavioral patterns during moral decision-making. J. Exp. Educ..

[29] Prehn K (2008). Individual differences in moral judgment competence influence neural correlates of socio-normative judgments. Soc. Cogn. Affect Neurosci..

[30] Perry JL, Clough PJ (2017). Predicting cooperation in competitive conditions: The role of sportspersonship, moral competence, and emotional intelligence. Psychol. Sport Exerc..

[31] Lind G, Fasko D, Willis W (2008). The meaning and measurement of moral judgment competence. A dual-aspect model. Contemporary Philosophical and Psychological Perspectives on Moral Development and Education.

[32] Dorrough AR, Glöckner A (2019). A cross-national analysis of sex differences in prisoner’s dilemma games. Br. J. Soc. Psychol..

[33] Haesevoets T, Bostyn DH, Reinders Folmer C, Roets A, van Hiel A (2019). Decision making in the prisoner’s dilemma game: The effect of exit on cooperation and social welfare. J. Behav. Decis. Mak..

[34] Mieth L, Buchner A, Bell R (2021). Moral labels increase cooperation and costly punishment in a Prisoner’s Dilemma game with punishment option. Sci. Rep..

[35] Malesza M (2020). The effects of the Dark Triad traits in prisoner’s dilemma game. Curr. Psychol..

[36] Strømland E, Tjøtta S, Torsvik G (2018). Mutual choice of partner and communication in a repeated prisoner’s dilemma. J. Behav. Exp. Econ..

[37] Taheri M, Rotshtein P, Beierholm U (2018). The effect of attachment and environmental manipulations on cooperative behavior in the prisoner’s dilemma game. PLoS ONE.

[38] Kuhn, S. Prisoner’s dilemma. *Stanford Encyclopedia of Philosophy* (2007).

[39] Balliet D, Mulder LB, van Lange PAM (2011). Reward, punishment, and cooperation: A meta-analysis. Psychol. Bull..

[40] Balconi M, Fronda G, Vanutelli ME (2019). A gift for gratitude and cooperative behavior: Brain and cognitive effects. Soc. Cogn. Affect Neurosci..

[41] Kim C-H (2005). Reciprocity in asymmetry: When does reciprocity work?. Int. Interact..

[42] Hoffman AJ (2020). The evolution of conflict, compassion and the social contract: A philosophical approach to human engagement. Aggress Violent Behav..

[43] Wood RI, Kim JY, Li GR (2016). Cooperation in rats playing the iterated prisoner’s dilemma game. Anim. Behav..

[44] Lin Z, Xu H, Fan S (2020). Evolutionary accumulated temptation game on small world networks. Phys. A.

[45] Janssen MA, Bushman C (2008). Evolution of cooperation and altruistic punishment when retaliation is possible. J. Theor. Biol..

[46] Stephens DW, McLinn CM, Stevens JR (2002). Discounting and reciprocity in an iterated prisoner’s dilemma. Science.

[47] Nowak MA, Sigmund K (2005). Evolution of indirect reciprocity. Nature.

[48] Sigmund K (2010). The Calculus of Selfishness.

[49] Kanazawa S, Fontaine L (2013). Intelligent people defect more in a one-shot prisoner’s dilemma game. J. Neurosci. Psychol. Econ..

[50] Cooper R, DeJong DV, Forsythe R, Ross TW (1996). Cooperation without Reputation: Experimental Evidence from Prisoner’s Dilemma Games. Games Econ. Behav..

[51] Gächter, S. Behavioral game theory. in *Blackwell Handbook of Judgment and Decision Making* 485–503 (Blackwell Publishing Ltd, 2004). 10.1002/9780470752937.ch24.

[52] Skinner BF (1953). Science and Human Behavior.

[53] Abide MM, Richards HC, Ramsay SG (2001). Moral reasoning and consistency of belief and behavior: Decisions about substance abuse. J. Drug Educ..

[54] Rholes WS, Bailey S (1983). The effects of level of moral reasoning on consistency between moral attitudes and related behaviors. Soc. Cogn..

[55] Breslin A (1982). Tolerance and moral reasoning among adolescents in Ireland. J. Moral Educ..

[56] Gächter S, Lee K, Sefton M (2020). Risk, Temptation, and Efficiency in Prisoner’s Dilemmas (No. 2020–15).

[57] Giamattei M, Yahosseini KS, Gächter S, Molleman L (2020). LIONESS Lab: A free web-based platform for conducting interactive experiments online. J. Econ. Sci. Assoc..

[58] Lind, G. Moral Competence Test (MCT). https://www.uni-konstanz.de/ag-moral/ (2020).

[59] SAS Institute, *SAS/STAT® 14.1 user’s guide* (2015).

